# Geometric characterisation of disease modules

**DOI:** 10.1007/s41109-018-0066-3

**Published:** 2018-06-18

**Authors:** Franziska Härtner, Miguel A. Andrade-Navarro, Gregorio Alanis-Lobato

**Affiliations:** 1 Faculty for Physics, Mathematics and Computer Science, Johannes Gutenberg Universität, Institute of Computer Science, Staudingerweg 7, Mainz, 55128 Germany; 2 Faculty of Biology, Johannes Gutenberg Universität, Institute of Molecular Biology, Ackermannweg 4, Mainz, 55128 Germany

**Keywords:** Protein interactions, Hyperbolic geometry, Disease modules, Greedy routing, Systems biology, Network medicine

## Abstract

There is an increasing accumulation of evidence supporting the existence of a hyperbolic geometry underlying the network representation of complex systems. In particular, it has been shown that the latent geometry of the human protein network (hPIN) captures biologically relevant information, leading to a meaningful visual representation of protein-protein interactions and translating challenging systems biology problems into measuring distances between proteins. Moreover, proteins can efficiently communicate with each other, without global knowledge of the hPIN structure, via a greedy routing (GR) process in which hyperbolic distances guide biological signals from source to target proteins.

It is thanks to this effective information routing throughout the hPIN that the cell operates, communicates with other cells and reacts to environmental changes. As a result, the malfunction of one or a few members of this intricate system can disturb its dynamics and derive in disease phenotypes. In fact, it is known that the proteins associated with a single disease agglomerate non-randomly in the same region of the hPIN, forming one or several connected components known as the disease module (DM).

Here, we present a geometric characterisation of DMs. First, we found that DM positions on the two-dimensional hyperbolic plane reflect their fragmentation and functional heterogeneity, rendering an informative picture of the cellular processes that the disease is affecting. Second, we used a distance-based dissimilarity measure to cluster DMs with shared clinical features. Finally, we took advantage of the GR strategy to study how defective proteins affect the transduction of signals throughout the hPIN.

**Electronic supplementary material:**

The online version of this article (10.1007/s41109-018-0066-3) contains supplementary material, which is available to authorized users.

## Introduction

Regardless of whether they represent the Internet or associations between proteins, people or airports; complex networks share many topological features (Albert and Barabási 2002), which suggests that similar rules govern their formation. Various models, aimed at mimicking the evolution and growth of these networks, assume the existence of a geometry underlying their structure and shaping their topology (Aste et al. 2005; Aste et al. 2012; Barthélemy 2011; Dall and Christensen 2002; Ferretti and Cortelezzi 2011; Krioukov et al. 2010; Papadopoulos et al. 2012). Recent work has shown that if such a geometry is hyperbolic, the emergence of the observed architecture of complex networks can be naturally explained by a distance-dependent connection probability between nodes in this metric space (Bianconi and Rahmede 2017; Ferretti et al. 2014; Krioukov et al. 2010; Papadopoulos et al. 2012; Wu et al. 2015).

In the native representation of complex networks in the two-dimensional hyperbolic plane $\mathbb {H}^{2}$, the *N* network nodes are enclosed inside a circle of radius *R*∼ ln*N*, each one lying at polar coordinates (*r*_*i*_,*θ*_*i*_) (Krioukov et al. 2010). These positions must ensure that connected nodes are close to each other and disconnected nodes are far apart. According to the definition of hyperbolic distance $d_{\mathbb {H}^{2}}(i, j) \approx r_{i} + r_{j} + 2\ln (\theta _{ij}/2)$ (Alanis-Lobato and Andrade-Navarro 2016; Krioukov et al. 2010; Papadopoulos et al. 2012), high-degree nodes are close to the centre of $\mathbb {H}^{2}$ because they need to be nearby many other nodes.

The embedding of real networks to hyperbolic space has shed light on their function and community organisation (Alanis-Lobato et al. 2018; Allard and Serrano 2018; Boguñá et al. 2010; García-Pérez et al. 2016; Serrano et al. 2012). For example, information routing overheads could be alleviated if the latent geometry of the Internet is used to guide packets between computers (Boguñá et al. 2010). Also, the geometry of the *E. coli* and human metabolic networks has put forward a new view of the definition of and interdependence between biochemical pathways (Serrano et al. 2012).

Of special interest for the present work is the analysis of the latent geometry of the human protein interaction network (hPIN). Alanis-Lobato and colleagues found that the hyperbolic map of the hPIN constitutes a meaningful and useful two-dimensional depiction of proteins and their interactions (Alanis-Lobato et al. 2018). The inferred radial coordinates of proteins hint at their evolutionary origin, whereas angular sectors group proteins with related biological functions and cellular localisations. In addition, hyperbolic distances can be used as likelihood scores for the prediction of biologically plausible protein-protein interactions (PPIs). Finally, Alanis-Lobato et al. showed that proteins can efficiently communicate with each other, without knowledge of the whole hPIN structure, by means of a greedy routing process in which hyperbolic distances guide biological signals from membrane receptors to transcription factors in the nucleus (Alanis-Lobato et al. 2018).

It is thanks to the effective transduction of signals throughout the hPIN that the cell operates, communicates with other cells and reacts to environmental stresses (Vinayagam et al. 2011). Therefore, defective or dysregulated proteins can disrupt PPIs, clog important signalling pathways and cause disease phenotypes (Taylor and Wrana 2012). In fact, it has been reported that the proteins associated with a single disease agglomerate non-randomly in the same region of the hPIN, forming one or several connected components known as the disease module (DM) (Agrawal et al. 2018; Menche et al. 2015). Consequently, disease-related proteins are more likely to have PPIs with each other than with random proteins. This particular connectivity pattern has been exploited to prioritise other proteins that may be related to a disease of interest (Cowen et al. 2017; Ghiassian et al. 2015; Köhler et al. 2008; Lage et al. 2007; Wu et al. 2008).

The above prompted us to analyse disease-associated proteins from a geometric perspective and to investigate how the latent geometry of the hPIN can reflect and expand our current knowledge about the organisation of DMs. We also took advantage of the greedy routing protocol to study the impact of disease proteins on the function of the hPIN.

## Results

### Topology and geometry of DMs

After the construction of a high-quality hPIN, its embedding to $\mathbb {H}^{2}$ (see Methods and Additional file 1: S1 and S2) and the evaluation of the embedding (see Methods, Additional file 2: Figures S1 and S2), we proceeded to analyse the topological and geometrical properties of DMs formed by the products of genes associated with 157 different diseases (see Methods and Additional file 1: S3).

**Fig. 1:**
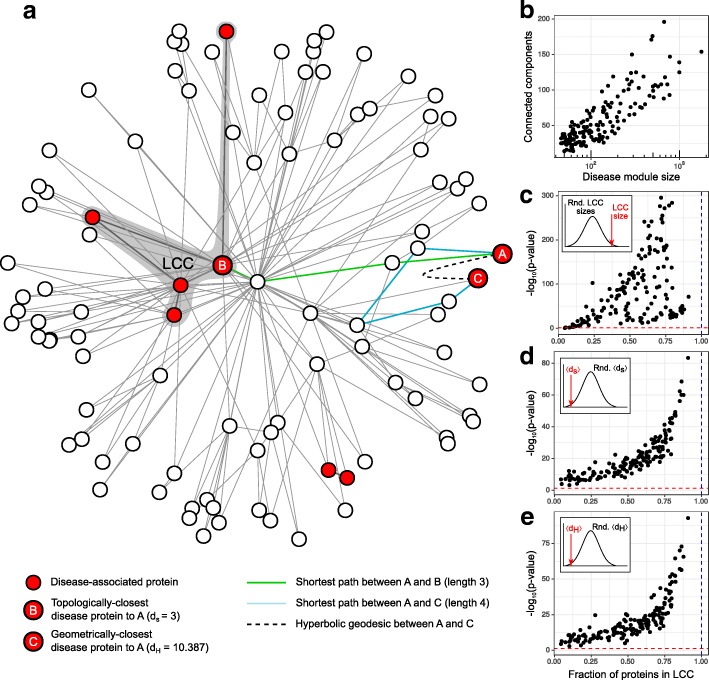
Topology and geometry of disease modules. **a** A toy protein network embedded in $\mathbb {H}^{2}$ and the gene products associated with a disease highlighted in red. The resulting DM is fragmented in 4 components and the largest one (LCC) has size 5. The topologically-closest protein to A is B (*d*_*s*_=3) and its geometrically-closest protein is C (*d*_*H*_=10.387), although it is 4 hops away from A. **b** The 157 studied DMs are split into several connected components in the hPIN. **c** The LCC size of each DM was compared with a random distribution of LCC sizes via a z-test. **d** The average of the shortest paths from each disease protein to its topologically-closest other DM member (〈*d*_*s*_〉) was compared with random expectation via a z-test. **e** The average of the hyperbolic distances from each disease protein to its geometrically-closest other DM member (〈*d*_*H*_〉) was compared with random expectation via a z-test. The red lines correspond to the significance level *α*=0.05 and the blue ones to the situation in which all proteins associated with a disease form a single connected component

In agreement with previous studies (Agrawal et al. 2018; Menche et al. 2015), we found that proteins associated with a single disease (see Fig. 1a) form several connected components in the hPIN, instead of a unique connected entity (see Fig. 1b). Nevertheless, the fraction of proteins contained in the largest of such components (LCC, see Fig. 1a) is significantly greater than expected by chance (see Methods and Fig. 1c). Besides, the average of the shortest paths from each disease protein to its closest other DM member (〈*d*_*s*_〉, see Fig. 1a) is smaller than expected by chance (see Methods and Fig. 1d), which indicates that, although DMs are fragmented, their components are made up of topologically nearby proteins (Menche et al. 2015).

To investigate how the above DM connectivity patterns are reflected in $\mathbb {H}^{2}$, we computed, for each of the *n* disease proteins in a DM, the hyperbolic distance to the closest other protein in the same module (*d*_*H*_, see Fig. 1a). The *n* resulting distances were averaged (〈*d*_*H*_〉) and compared with a distribution of average hyperbolic distances resulting from randomly sampling as many proteins from the hPIN as there are in each considered DM (see Methods). Figure 1e shows that, in all 157 cases, 〈*d*_*H*_〉 is significantly shorter than expected by chance. This result points out that in $\mathbb {H}^{2}$ DMs are fragmented in components formed by at least two hyperbolically nearby proteins. While this appears to be a predictable result, it is important to note that the DM member that is topologically-closest to a protein in the same module can be different from the geometrically-closest one (see Fig. 1a).

### DMs are formed by functionally distinct submodules

The observed level of DM fragmentation has been attributed to the incompleteness of the hPIN and the limited knowledge of disease genes (Menche et al. 2015). While these two factors may certainly contribute to DM splitting, disease-related genes belong to broad functional categories (Jimenez-Sanchez et al. 2001) and locate to distant cellular compartments, which makes their PPI unlikely (Thul et al. 2017). As such, DMs are most probably formed by functionally distinct submodules that, together, contribute to the disease phenotype.

**Fig. 2:**
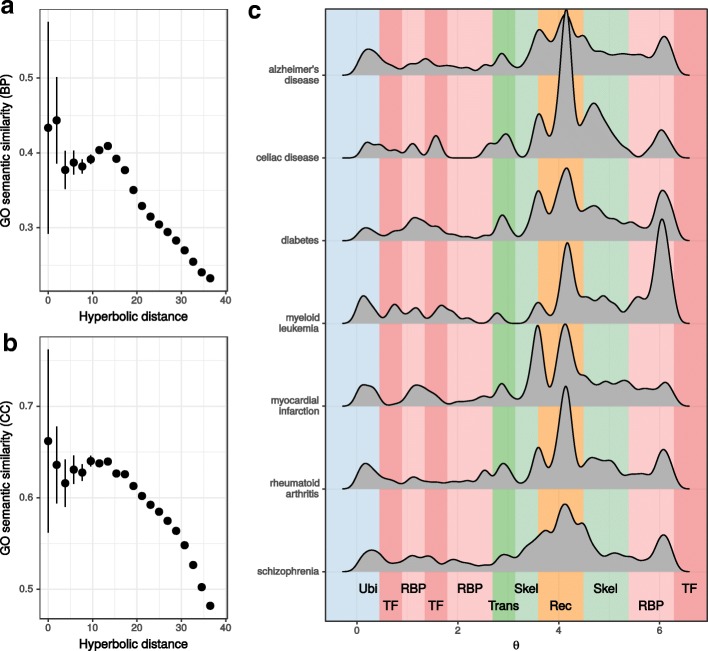
Disease modules split into functionally distinct submodules. **a**. Gene Ontology (GO) semantic similarity of Biological Process (BP) terms that annotate proteins pairs spaced not further than the indicated hyperbolic distance. **b**. Same as **a** but for Cellular Component (CC) terms. **c** Angular distribution of disease proteins associated with 7 illnesses from our gene-disease association dataset. The coloured backgrounds indicate that one of 6 considered protein classes is over-represented in that angular range (Ubi: proteins involved in ubiquitination/proteolysis, TF: transcription factors, RBP: RNA-binding proteins, Trans: transporters, Skel: constituents of the cytoskeleton, Rec: receptors)

In $\mathbb {H}^{2}$, neighbouring proteins play roles in very similar biological processes and lie in the same organelles (see Fig. 2a, b and Methods). Furthermore, Alanis-Lobato et al. reported that the angular component of hyperbolic space captures the functional and spatial organisation of the cell (Alanis-Lobato et al. 2018) (see Additional file 2: Figure S2b). Therefore, we partitioned the angular dimension into several sectors, according to the over-represented protein class within them (see Methods), and studied whether the distribution of inferred angular coordinates of disease proteins does hint at their functional modularity.

Figure 2c and Additional file 1: S3 promptly highlight the protein class heterogeneity of DMs, together with interesting correlations between protein function and features of the disease. For example, almost all diseases exhibit a strong receptor (Rec) component. Since most drug-targets are membrane Recs, this could be the result of study biases against membrane proteins (Brito and Andrews 2011). However, this also points at the crucial role of signalling in cell operation (Vinayagam et al. 2011): celiac disease and rheumatoid arthritis, the DMs with the highest peaks at the Rec-enriched sector, are both characterised by altered inflammatory signalling pathways (Coenen et al. 2009). On the other hand, a high peak in the sector enriched for RNA-binding proteins (RBPs) may be indicative of problems in post-transcriptional gene regulation (Lukong et al. 2008). In fact, myelodysplastic syndromes have been linked with the malfunction of different components of the spliceosome and frequently develop into myeloid leukaemia, the DM with the highest RBP-related peak (Turner and Monzón-Casanova 2017).

### Distance-based clustering of DMs

A pathophysiology-based classification system of human disorders is pivotal in diagnosis and research (Baird 2013). The International Statistical Classification of Diseases and Related Health Problems 10th Revision (ICD-10) is an effort of the World Health Organisation to categorise morbid entities according to established criteria (WHO 2016). Despite the fact that, in practice, the ICD has become the standard classification, it has to be periodically revised (WHO 2016). Revisions remain a challenging task: categories must have a strong scientific basis, many diseases seem to be a complicated mix of disorders and many lack archetypal biomarkers, which means that their diagnosis still depends on subjective criteria (Baird 2013). Could DM features aid in this task?

Menche et al. introduced a measure of topological overlap between DMs to identify their shared clinical characteristics (Menche et al. 2015). This measure of separation compares the average shortest path between each DM member and the nearest protein within the module, 〈*d*_*s*_(*A*,*A*)〉 and 〈*d*_*s*_(*B*,*B*)〉, to the average shortest path between each DM member and the nearest protein in the other module, 〈*d*_*s*_(*A*,*B*)〉: *s*_*s*_(*A*,*B*)=〈*d*_*s*_(*A*,*B*)〉−(〈*d*_*s*_(*A*,*A*)〉−〈*d*_*s*_(*B*,*B*)〉)/2. We translated this definition to the geometric position of DMs in $\mathbb {H}^{2}$ by considering the average hyperbolic distance between each DM member and the closest protein in the same, 〈*d*_*H*_(*A*,*A*)〉, or another module, 〈*d*_*H*_(*A*,*B*)〉 (see Methods).

**Fig. 3:**
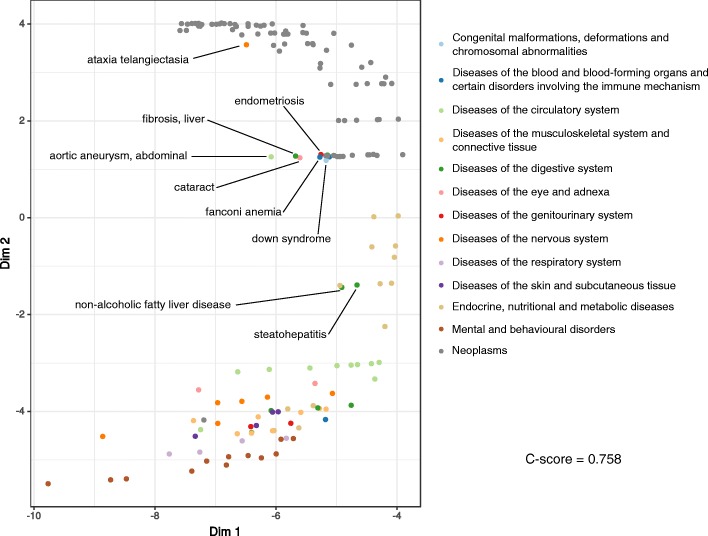
Distance-based clustering of disease modules. ncMCE, a nonlinear dimensionality reduction algorithm, was applied to the matrix of pairwise hyperbolic distance-based DM separations. The resulting two-dimensional projection of the data separates DMs according to the disease type reported in the ICD-10. Some interesting DMs that were not correctly clustered are highlighted. The concordance score (C-score) over Dim 2 for this projection is 0.758. The points corresponding to *endometriosis* and *down syndrome* were slightly moved to make them visible

We used the distance-based separation between DMs, *s*_*H*_(*A*,*B*), to construct a matrix of pairwise module separations. Then, we resorted to non-centred Minimum Curvilinear Embedding (ncMCE) for the non-linear dimensionality reduction of our 157 DMs through this matrix (Cannistraci et al. 2013) (see Methods). Figure 3 shows that the use of *s*_*H*_(*A*,*B*) leads to the distinction of ICD-10 categories that group pathobiologically and clinically similar illnesses. This is in spite of the observed fragmentation and functional heterogeneity of DMs (see Figs. 1 and 2). We quantified the quality of the resulting clustering with the concordance score (C-score, see Methods) and found that it is close to what *s*_*s*_(*A*,*B*) achieves and better than using just intersections between the members of DMs (see Fig. 3, Additional file 2: Figure S4 and the Methods). Moreover, using the *s*_*H*_(*A*,*B*) matrix as the basis for a hierarchical clustering of DMs recapitulates the disease types exposed by ncMCE (see Additional file 2: Figure S5 and the Methods).

Figure 3 also highlights some DMs whose ICD-10 classification does not correspond to their ncMCE cluster. Yet, we found reasonable explanations for all these DMs. For example, non-alcoholic fatty liver disease and steatohepatitis (a more extreme variety of the former) are both strongly associated with insulin resistance and metabolic syndromes (Tolman and Dalpiaz 2007), which explains why they are clustered with endocrine and metabolic disorders (ICD-10 classifies them as diseases of the digestive system).

The other cases involve conditions clustered with cancers but categorised differently by the ICD-10. Ataxia telangiectasia (AT) is considered a neurodegenerative disease but it is linked to a high risk for cancer (Gilmore 2014). Mutations in the gene coding for the kinase ATM were identified as the main cause of AT (Gilmore 2014). ATM is mainly involved in the recruitment of DNA repair complexes in response to single or double strand breaks, hence the relationship between AT and neoplasms (Cremona and Behrens 2013).

Endometriosis is a disease characterised by the presence of endometrial-like tissue outside the uterine cavity, causing a chronic inflammatory response (Heidemann et al. 2013). Women with atypical endometriosis have a significantly increased risk of certain forms of ovarian cancer. Likewise, women with ovarian cancer are more likely to have the disease (Vercellini et al. 2013). In addition, there is also increasing awareness that women with endometriosis have a higher risk for developing breast cancer (Roy et al. 2015; Pontikaki et al. 2015).

Liver fibrosis is the result of constant inflammation of the liver, leading to excessive accumulation of extracellular matrix proteins, especially collagen. Severe liver fibrosis can lead to hepatocellular carcinoma (Uehara et al. 2013; Sakurai and Kudo 2013).

Down syndrome (DS) is known to have several associated haemopoietic conditions, leukaemia amongst them. Children with DS have a 10-20 times higher risk of having leukaemia than non-DS children (Bruwier and Chantrain 2011; Xavier et al. 2009).

Fanconi anaemia (FA) is a rare bone marrow failure disease leading to impaired DNA repair response. This disease also leads to haematologic changes. So far, mutations in 19 DNA damage response genes are known to either cause FA or increase the risk to have it (Bogliolo and Surrallés 2015). Depending on the affected genes, FA increases the risks for developing leukaemia, solid tumours, breast and ovarian cancer (Alter 2014; Bogliolo and Surrallés 2015).

Finally, we did not find conclusive associations between abdominal aortic aneurysm and cataracts with neoplasms. However, smoking is the strongest risk factor of the former (Kent 2014), which could explain its concomitance with lung cancer (Blochle et al. 2008). For the latter, the association between early-onset cataracts and insufficient anti-oxidative activity inspired a study in which early-onset cataract and healthy patients were followed for several years to estimate the incidences of cancer. The result was a two-fold higher cancer risk for the early-onset cataract cohort (Chiang et al. 2014).

### Impact of disease proteins on cellular function

Signal transduction is the process of translating external signals at the cell membrane to specific responses within the cell. In this manner, cells react to environmental stresses by switching on certain genes and turning off others (Vinayagam et al. 2011). Interestingly, membrane receptors are only aware of their direct interaction partners, not the entire hPIN structure. Nonetheless, they manage to relay an external signal to the appropriate neighbour, so that it reaches transcription factors (TFs) in the nucleus that up-regulate target genes (Vinayagam et al. 2011).

The efficiency of signal transduction throughout the hPIN can be studied by means of its latent geometry and a navigation strategy known as greedy routing (GR) (Kleinberg 2000). In GR, inferred node coordinates are used as addresses that guide a signal from a source to a target node. The source checks amongst its direct neighbours for the hyperbolically closest to the target and sends the signal there, the recipient node does the same and the process is repeated until the signal reaches the target. If the signal is sent to a previously visited node (i.e. it falls into a loop), the routing is considered unsuccessful (Boguñá et al. 2009; Ortiz et al. 2017; Papadopoulos et al. 2010). GR efficiency (i.e. network navigability) is quantified as the fraction of successful routing events from a sufficiently large number of randomly chosen source-target pairs (Krioukov et al. 2010).

**Fig. 4:**
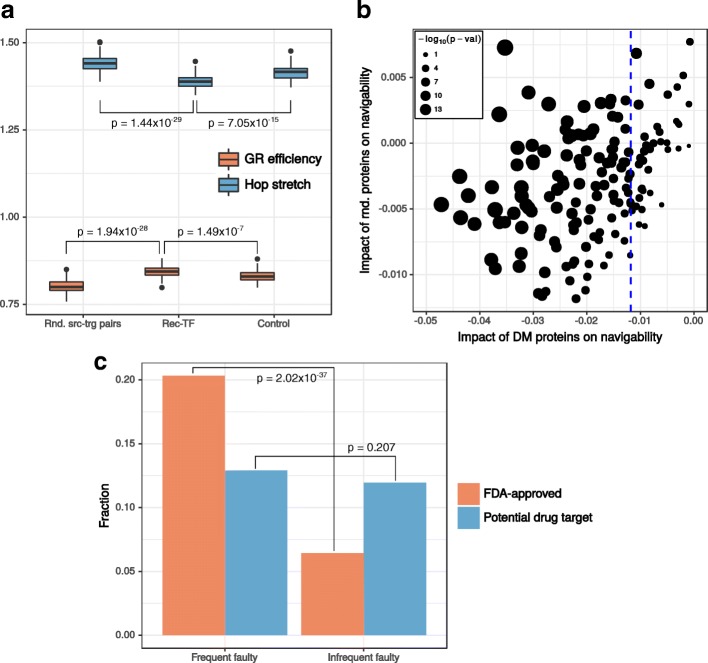
Disease proteins affect signal transduction. **a** Reference greedy routing (GR) efficiency and hop stretch of the hPIN (Rnd. src-trg pairs) compared to the efficiency (hop stretch) achieved if signals are routed between receptors (Rec) and transcription factors (TF) or between proteins that are neither Recs nor TFs, but that have degrees similar to their counterparts (Control). **b** Impact on GR efficiency (navigability) dealt by faulty proteins sampled from the set of proteins associated with a DM or from a pool of proteins with similar degrees. The blue dashed line indicates the value of the highest impact (i.e. the most negative) from the latter case. **c** Frequently used faulty proteins are more likely to be FDA-approved drug targets or potential drug targets than infrequent faulty proteins. *P*-values are the result of a Fisher’s test comparing the proportion of FDA-approved or potential drug targets in frequent vs infrequent faulty proteins

We measured the reference efficiency of the hPIN by considering 500 source-target pairs and repeating the measurement 100 times. Figure 4a shows that the median GR efficiency of the hPIN is 0.8 and the median hop stretch (length of the greedy path between two nodes divided by length of their shortest path) is close to 1. This figure also highlights the biological importance of signal transduction: GR efficiency (hop stretch) for Rec-TF pairs is significantly bigger (smaller) than the reference and what is achieved by considering proteins that are neither Recs nor TFs, but have degrees similar to their counterparts (see Additional file 2: Figure S6 and the Methods).

The communication channels between Recs and TFs are so important that almost all known diseases are linked to their dysfunction (Thul et al. 2017). We studied the impact of disease proteins on GR efficiency through the introduction of faulty proteins to the GR process (Alanis-Lobato et al. 2018). Faulty proteins drop any signals they receive, making routing unsuccessful, and model the effects caused by mutations or insufficient protein levels. For each DM, we measured the average of 50 GR efficiencies resulting from routing signals between 500 source-target pairs. Based on a study estimating 3-24 homozygous disease-causing mutations per individual (Xue et al. 2012), we introduced 20 faulty proteins to each one of the 50 experiments. Faulty proteins were sampled from the members of a DM or chosen at random from a pool of proteins with similar degrees. In each case, we quantified the impact on navigability as the difference between the resulting average efficiency and the reference efficiency of the hPIN (0.82). The more negative the difference, the higher the impact (see Methods).

Figure 4b shows that disease proteins deal a higher impact on network navigability than faulty proteins sampled randomly from the hPIN (see Methods). Besides, for 89.17*%* of the DMs, this impact is significantly greater than expected (see Methods). Interestingly, the functional enrichment analysis (see Methods) of proteins associated with these DMs (mostly neoplasms and diseases of the nervous system) revealed that they are mainly receptors whose failure affects gene transcription and apoptosis (see Additional file 2: Figure S7a). By contrast, proteins associated with the remaining 10.83*%* DMs (mostly diseases of the connective tissue and metabolic disorders) are receptors whose failure affects inflammatory responses and cell homeostasis (see Additional file 2: Figure S7b). These results further support our assumption that GR simulates signal transduction events from Recs to TFs.

Given the high percentage of DMs whose members exert a negative impact on GR, recurring gene products in the lists of faulty proteins from the above experiments could hint at the biological role played by proteins responsible for such an impact. We focused on faulty proteins reappearing more than the upper quartile of the frequency distribution (frequent faulty) and found that they are mostly the products of genes with enzymatic and receptor activity (see Additional file 2: Figure S8a), whereas infrequent faulty proteins are involved in more heterogeneous functions (see Additional file 2: Figure S8b). Since current FDA-approved drugs are mainly directed to enzymes and receptors (Brito and Andrews 2011; Thul et al. 2017), we reasoned that this methodology could be used to identify potential drug targets. Indeed, a significant fraction of frequent faulty proteins are reported FDA-approved drug targets (see Fig. 4c, Methods and Additional file 1: S4). Also, although not significant, the fraction of frequent faulty proteins that are considered potential drug targets by the Human Protein Atlas (Thul et al. 2017) is higher than the fraction of infrequent faulty proteins with the same property (see Fig. 4c and Additional file 1: S4).

Potential drug targets are proteins whose structure, biochemical aspects and associated pathways make them candidate druggable proteins (Thul et al. 2017). Intriguingly, some of the most frequent faulty proteins that we identified are already being investigated for their therapeutic potential, which endorses the value of the proposed GR-based approach for drug target prioritisation (see Additional file 1: S4). For example, Fienberg and colleagues are trying to selectively inhibit the domains of the ACE protein to treat fibrosis and hypertension (Fienberg et al. 2018); Ding et al. are developing dual inhibitors of EGFR and PI3K *α* as an approach against tumours (Ding et al. 2018); similarly, early-phase trials suggest that therapies targeting the PI3K/AKT/mTOR pathway can be used in patients with advanced cancers (Janku et al. 2013). Finally, it has been shown that directly targeting STAT3 with piperlongumine has positive and potent effects against breast cancer (Bharadwaj et al. 2014).

## Conclusions

The representation of the human protein interaction network in the two-dimensional hyperbolic plane has been shown to be both meaningful and useful: inferred node coordinates convey information about protein evolution and function, whereas hyperbolic distances can be used to identify potential protein interactions and simulate signalling events (Alanis-Lobato et al. 2018).

In this paper, we report yet another scenario in which the latent geometry of the hPIN proves useful, namely the network-based analysis of disease-associated proteins. First, we found that the geometric position of disease modules reflects their fragmentation and functional heterogeneity, underscoring the complexity of human disorders. Visualisation of the angular distribution of DM members, together with the protein classes over-represented in different sectors of $\mathbb {H}^{2}$, renders an informative picture of the cellular processes that the disease is affecting. Second, we used a hyperbolic distance-based dissimilarity measure to cluster DMs. The resulting clusters are in good agreement with the standard ICD-10 and bring out unexpected but reasonable relationships between disorders. Finally, we studied how defective proteins affect the efficient routing of signals throughout the hPIN. Interestingly, proteins that were frequently considered faulty in our experiments represent known or potential drug targets.

While our geometric characterisation of DMs was carried out on a proteome-scale high-quality network, human PPI maps are still incomplete (Luck et al. 2017). In consequence, the projection to $\mathbb {H}^{2}$ can change if more interactions or proteins are considered. To test the robustness of our findings to network topology changes, we repeated our experiments on an independent PPI dataset and observed the same trends (see Additional file 2: Figures S11–S14, Additional file 1: S5-S6 and the Methods). The consistency between the two independent analyses reasserts the validity of our results.

Improvements in the sensitivity and scalability of PPI detection methods will ultimately lead to a more complete picture of the network of protein interactions that take place in the human cell (Luck et al. 2017). This reference PPI map will allow for a more accurate depiction of the network in hyperbolic space, which in turn will represent a more powerful tool for the analysis of protein and cellular functions. Based on the present study, we anticipate that the integration of the hPIN geometry with gene-disease association data will play a key role in advancing our understanding of human disease and defining the druggable proteome.

## Methods

### Gene-disease associations

We obtained gene-disease associations from DisGeNET v5.0 (Piñero et al. 2017), filtered out gene-disease pairs supported by less than 3 publications and diseases with less than 50 associated genes. To avoid redundancies, we merged diseases with very similar lists of associated genes. For this, we constructed a Jaccard similarity matrix between diseases to cluster them hierarchically. Upon inspection of the resulting dendrogram, we merged diseases that ended up in the same cluster after cutting the tree at height 6. If *X* and *Y* represent the sets of genes associated with two different diseases, their Jaccard similarity is obtained with *J*(*X*,*Y*)=|*X*∩*Y*|/|*X*∪*Y*| (Jaccard 1912).

The described filtering and merging process resulted in 157 diseases. We assigned them to their corresponding disease type using Revision 10 of the ICD (WHO 2016).

### Construction of the hPIN

We built the hPIN with high-quality interactions from the Human Integrated Protein-Protein Interaction rEference (HIPPIE) v2.0 (Alanis-Lobato et al. 2017). Each PPI in HIPPIE has an assigned confidence score. Based on reference estimates of the hPIN size (Venkatesan et al. 2009), we considered the 155,000 interactions with the highest scores. After discarding self-interactions, merging redundant PPIs by considering their maximum score and extracting the network’s LCC, we finally arrived to an hPIN formed by 150,212 interactions between 14,788 proteins. Protein names were translated to their corresponding Entrez ID and gene symbol with the MyGene.info web service (Xin et al. 2016).

In addition, we verified the validity of our results in an independent network dataset: the LCC formed by PPIs from the HINT database (19/02/18 snapshot of binary interactions) (Das and Yu 2012). The HINT network is comprised of 47,181 interactions between 10,370 proteins.

### Identification of proteins classes and drug targets

We integrated information from several resources to identify proteins with TF, receptor, transporter or RNA-binding activity; as well as constituents of the cytoskeleton and proteins involved in ubiquitination/proteolysis. Our pool of TFs comes from the Animal Transcription Factor Database 2.0 (Zhang et al. 2015), the census of human TFs (Vaquerizas et al. 2009) and the Human Protein Atlas (Uhlen et al. 2015). From the latter, we also collected constituents of the cytoskeleton, proteolysis- and cancer-related proteins, receptors, transporters, RBPs, FDA-approved and potential drug targets. Additional receptors and transporters were taken from the Guide to Pharmacology (Southan et al. 2015). We also took into account RBPs from the RBP census (Gerstberger et al. 2014).

### Mapping the hPIN to hyperbolic space

We mapped the hPIN to the two-dimensional hyperbolic plane using LaBNE+HM (Alanis-Lobato et al. 2016b), a method that combines manifold learning (Alanis-Lobato et al. 2016a) and maximum likelihood estimation (Papadopoulos et al. 2015) for fast yet accurate embeddings. We used the method implemented in R’s package NetHypGeom (https://github.com/galanisl/NetHypGeom) with parameters *γ*=2.644, *T*=0.827 and *w*=2*π*. We mapped the HINT network to $\mathbb {H}^{2}$ using the same method with parameters *γ*=2.115, *T*=0.882 and *w*=2*π*.

### Evaluation of the hyperbolic map

In order to ensure that the inferred geometry of the hPIN and HINT was compatible with their network topology, we considered four network-structure-related criteria: (i) We divided the range of hyperbolic distances between proteins into 20 bins and, for each one, computed the fraction of protein pairs that are connected in the network (i.e. the connection probability for a distance window). We checked if these empirical connection probabilities agree with those predicted by the Popularity-Similarity model (PSM). The PSM is a model of network formation in which nodes connect with each other if their hyperbolic distance is small enough (Papadopoulos et al. 2012). (ii) We checked if expected node degrees $\langle k_{i} \rangle = \sum _{j\neq i} p_{ij}$ were similar to actual node degrees. $p_{ij} = 1/\left [1 + e^{(x_{ij} - R)/2T}\right ]$ is the probability that node *i* forms a link with node *j* and depends on the hyperbolic distance *x*_*ij*_ described in the Introduction. *R* is the radius of the hyperbolic disc containing the network and *T* is the network temperature. (iii) We checked, if the clustering coefficient of the protein networks was similar to the average clustering of 10 artificial networks generated with the PSM, using the same topological properties of the hPIN and HINT. (iv) Finally, we checked whether greedy routing success rates and hop stretches were similar to those achieved in artificial networks generated with the PSM, using the same topological properties of the hPIN and HINT.

In addition, we validated the biological interpretation that Alanis-Lobato and colleagues reported for inferred protein coordinates (Alanis-Lobato et al. 2018). For this, we assigned all network proteins to six different age groups using FastaHerder2 (Mier and Andrade-Navarro 2016) and looked at the distribution of inferred radial coordinates for each one. Also, we studied how proteins from different classes (i.e. TFs, Recs, RBPs, etc.) agglomerate in the angular dimension of $\mathbb {H}^{2}$.

### Statistical tests for DM topology and geometry

Using a z-test and a significance level *α*=0.05, we compared the size of the LCC that each disease module forms in the hPIN with a distribution of 1,000 random LCC sizes formed by as many proteins as there are in each module, but sampled uniformly at random from the set of all network nodes (see inset in Fig. 1c).

For each of the *n* disease proteins in a DM, we determined the shortest path and hyperbolic distance to the closest other protein in the same module (*d*_*s*_ and *d*_*H*_, respectively). The *n* resulting distances were averaged (〈*d*_*s*_〉 and 〈*d*_*H*_〉) and compared with a distribution of 1000 average *d*_*s*_ and *d*_*H*_ resulting from randomly sampling as many proteins from the hPIN as there are in each considered DM (see insets in Fig. 1d, e). A z-test and a significance level *α*=0.05 were employed in both cases.

### GO semantic similarities

We divided the range of hyperbolic distances between proteins into 20 bins and, for each one, computed the average Gene Ontology (GO) semantic similarity of protein pairs within the distance window. GO semantic similarities are a valuable means to quantify the level of similitude between the GO annotations associated with two genes. We used the R package GOSemSim (Yu et al. 2010) to calculate Wang similarities from the Biological Process and Cellular Compartment aspects of GO. We decided to use Wang’s index because it was formulated specifically for the GO (Wang et al. 2007).

### Protein classes in the angular dimension

We split the range of inferred protein angular coordinates into 15 bins and, for each one, we carried out six Fisher’s tests to determine the most over-represented protein class in the bin from the six considered classes (see above). If two or more adjacent bins were enriched for the same protein class, the whole range covered by the bins was regarded as representative for that class. We chose 15 bins as it was the minimum number necessary for all the 6 protein classes to be represented.

### Disease module separation and clustering

The separation between two disease modules *A* and *B* in hyperbolic space is inspired by the shortest path-based measure proposed by Menche and colleagues (Menche et al. 2015). The separation is defined as follows:

$$s_{H}(A,B) = \langle d_{H}(A,B) \rangle - \frac{\langle d_{H}(A,A) \rangle + \langle d_{H}(B,B) \rangle}{2} $$

The more negative *s*_*H*_(*A*,*B*), the greater the overlap between modules A and B. We compared this distance-based measure with the original shortest path-based one *s*_*s*_(*A*,*B*) and with the Jaccard distance between DMs. The latter compares the size of the intersection between the members of two modules to the size of their union: *J*(*A*,*B*)=1−|*A*∩*B*|/|*A*∪*B*| (Jaccard 1912).

The resulting pairwise separations and Jaccard distances between DMs were used for their unsupervised classification via ncMCE and hierarchical clustering with Ward’s linkage (Ward Jr 1963). We used ncMCE, a non-linear dimensionality reduction algorithm, because it is parameter-free and excels at emphasising small differences between samples. Its two dimensional projections cluster similar observations along the *y*-axis and outline their diversity on *x* (Alanis-Lobato et al. 2015; Cannistraci et al. 2013). To measure the ability of ncMCE to separate DMs into reference categories (ICD-10) along the *y*-axis, we employed the C-score. The C-score ranges from 0 to 1, where 0 corresponds to a completely random clustering and 1 to a perfect ordering of samples along a single dimension, i.e. disease types appear one after the other with no DMs belonging to one, mixed with the other (Alanis-Lobato et al. 2015; Zagar et al. 2011).

### Navigability impact of faulty proteins

GR of signals involved 100 experiments with 500 sources and 500 targets each. Sources and targets were selected at random from the hPIN or from a pool of Recs or TFs. We used pools with the same amount of Recs and TFs (500 randomly-selected) to make sure that the observed effects were not due to different abundances of these protein types in the hPIN. In addition, we formed pools of non-Recs and non-TFs with degrees similar to the ones exhibited by the actual members of each class (Control experiments). The resulting Rec-TF efficiencies and hop stretches were compared to reference and Control via Mann-Whitney U tests.

For each DM, we calculated the impact on navigability as the average of the 50 efficiencies resulting from routing signals between 500 source-target pairs (with 20 faulty proteins sampled from the set of DM members) minus the reference average efficiency of the hPIN (0.82). The same was done for faulty proteins sampled from a pool of proteins with degrees similar to the DM members’. To assess if the difference between both cases was significant, we computed the 50 impacts separately, compared them with a Mann-Whitney U test and considered a significance level *α*=0.05.

GO and REACTOME enrichment analyses were carried out with R’s package FunEnrich (https://github.com/galanisl/FunEnrich). *P*-values were corrected with Benjamini-Hochberg’s method.

To compare the proportion of frequent faulty proteins that are FDA-approved or potential drug targets with the proportions in the infrequent faulty protein set, we used a Fisher’s test and considered a significance level *α*=0.05.

### Hardware used for experiments

We executed the experiments presented in this paper on a Lenovo ThinkPad 64-bit with 7.7 GB of RAM and an Intel Core i7-4600U CPU @ 2.10 GHz × 4, running Ubuntu 16.04 LTS. The only exceptions were the greedy routing experiments, which we executed on nodes with 30 GB of RAM, within the Mogon computer cluster at the Johannes Gutenberg Universität in Mainz.

## Additional files

Additional file 1 Supplementary files. (ZIP 3154 kb)

Additional file 2 Supplementary information. (PDF 5687 kb)
