# Supplementary material for: Geometric characterisation of disease modules
Source: Appl Netw Sci. 2018 Jun 18;3(1):10. doi: 10.1007/s41109-018-0066-3 (PMC6214295; doi:10.1007/s41109-018-0066-3)
Supplement: Supplementary file 2 — Supplementary information. (PDF 5687 kb) [file 41109_2018_66_MOESM2_ESM.pdf]

SUPPLEMENTARY INFORMATION

Geometric characterisation of disease modules

Franziska Härtner[1], Miguel A. Andrade-Navarro[2], and Gregorio Alanis-Lobato[*,2]

[1] *Faculty for Physics, Mathematics and Computer Science, Johannes Gutenberg Universität, Institute of Computer Science, Staudingerweg 7, 55128 Mainz, Germany*
[2] *Faculty of Biology, Johannes Gutenberg Universität, Institute of Molecular Biology, Ackermannweg 4, 55128 Mainz, Germany*

[*] Correspondence should be addressed to galanisl@uni-mainz.de

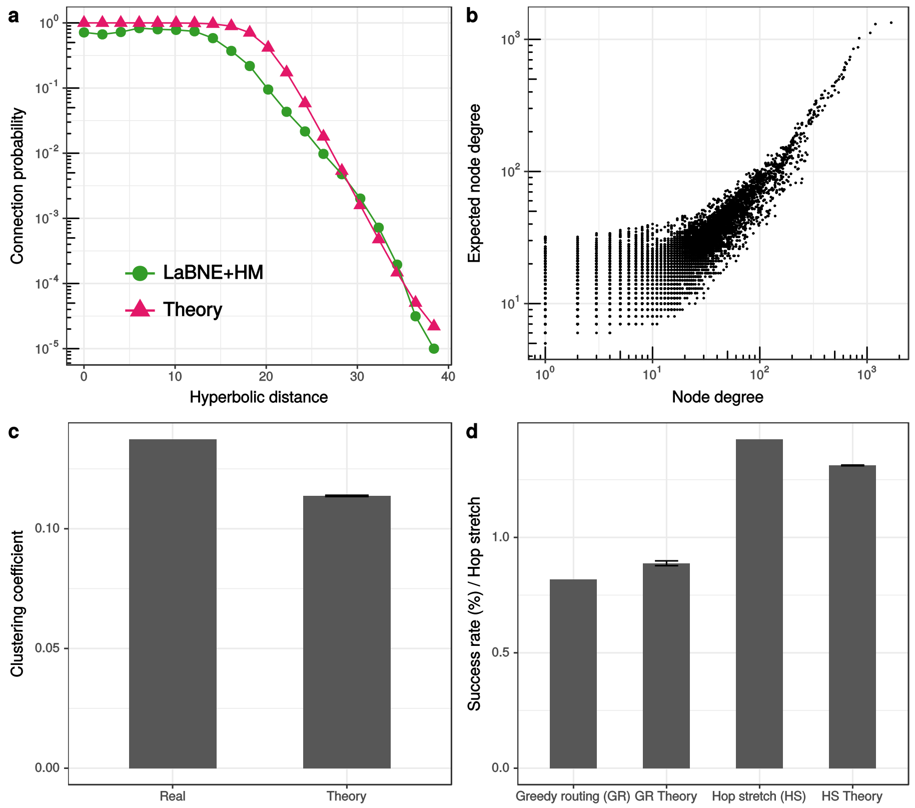

Figure S1: Topological evaluation of the hPIN embedding. The quality of the hyperbolic embedding of the considered protein network (hPIN) was assessed with four different criteria: **(a)** Empirical connection probabilities (LaBNE+HM) agree with those predicted by the Popularity-Similarity model (PSM, Theory). **(b)** Expected node degrees $\langle k_i \rangle = \sum_{j \neq i} p_{ij}$ are similar to actual node degrees. $p_{ij} = 1/[1 + e^{(x_{ij} - R)/2T}]$ is the probability that node i forms a link with node j and depends on the hyperbolic distance x_{ij} between them. R is the radius of the hyperbolic disc containing the network and T is the network temperature. **(c)** The clustering coefficient of the hPIN is similar to the clustering of artificial networks generated with the PSM, using the same topological properties of the hPIN (the average across 10 PSMs is reported, errors bars correspond to standard deviations). **(d)** Greedy routing success rates and hop stretches are similar those achieved in artificial networks generated with the PSM, using the same topological properties of the hPIN (the average across 10 PSMs is reported, errors bars correspond to standard deviations).

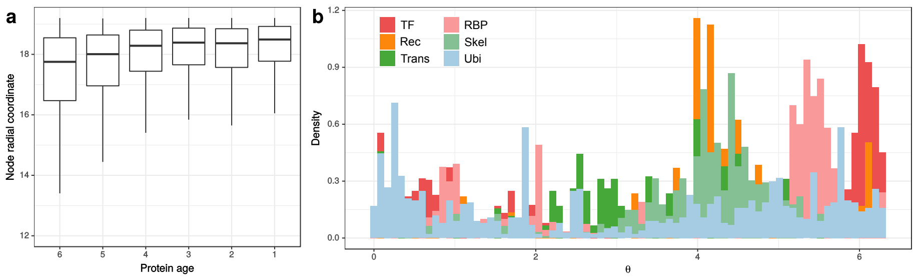

Figure S2: Biological evaluation of the hPIN embedding. **(a)** Old proteins tend to be close to the centre of hyperbolic space, while young proteins lie on its periphery. Age groups correspond to human proteins also present in 6: Cellular organisms, 5: Metazoa, 4: Chordata, 3: Mammalia, 2: Euarchontoglires, 1: Primates. **(b)** The inferred angular coordinates of proteins capture the functional organisation of the cell.

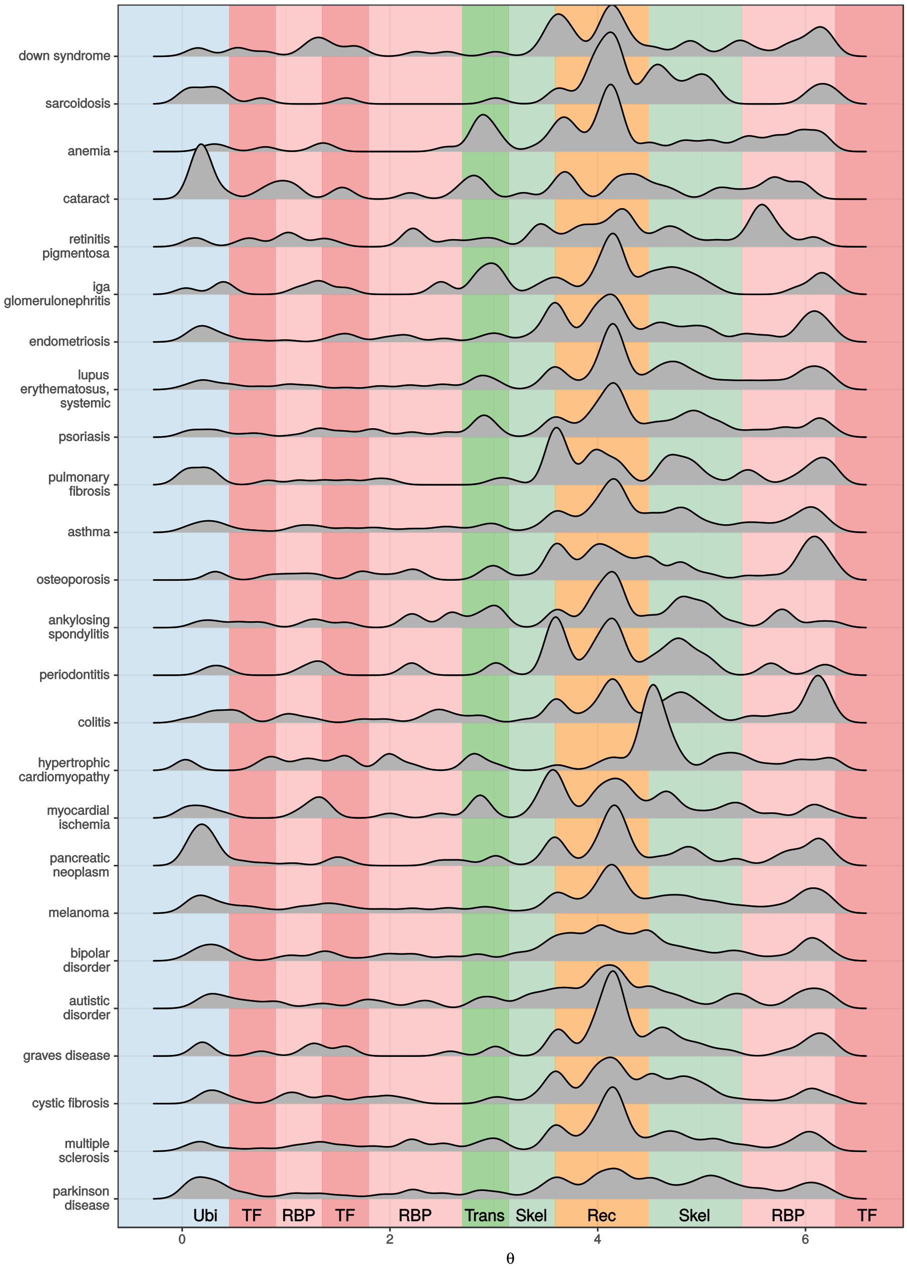

Figure S3: Disease modules split into functionally distinct submodules. Angular distribution of disease proteins associated with 25 illnesses from our gene-disease association dataset. The plot highlights the protein class heterogeneity of disease modules (Ubi: proteins involved in ubiquitination/proteolysis, TF: transcription factors, RBP: RNA-binding proteins, Trans: transporters, Skel: constituents of the cytoskeleton, Rec: receptors).

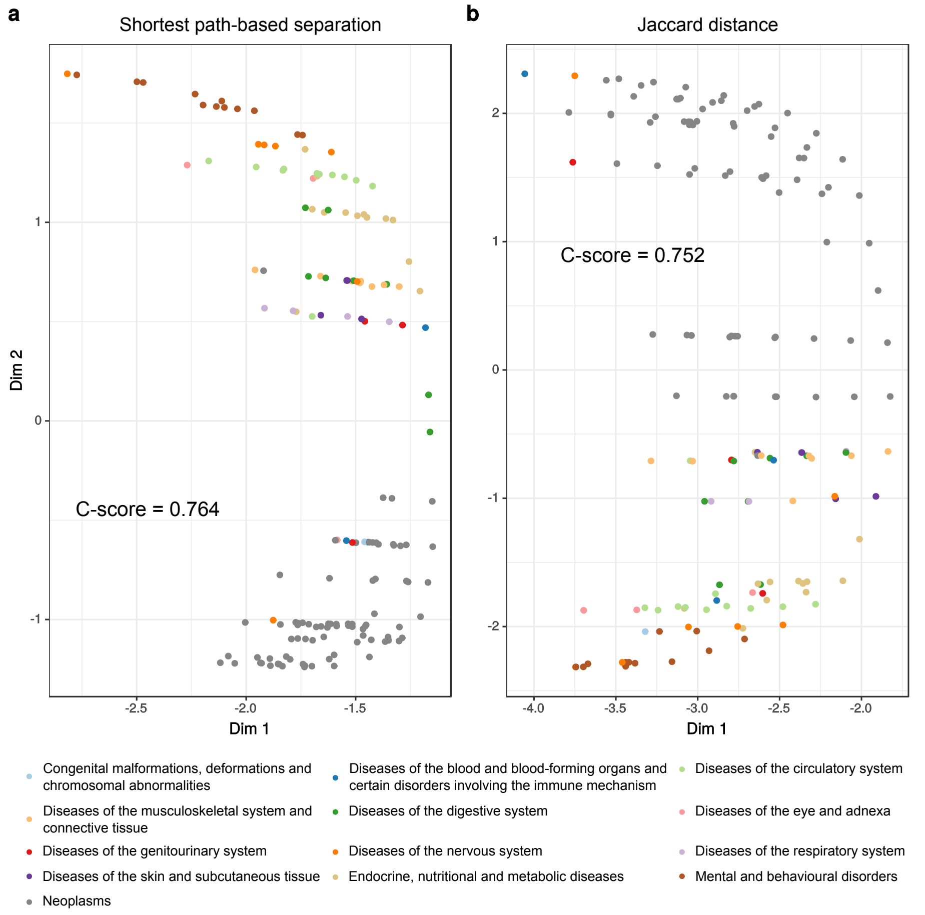

Figure S4: Clustering of DMs (a) ncMCE, a nonlinear dimensionality reduction algorithm, was applied to the matrix of pairwise shortest path-based DM separations. The resulting two-dimensional projection of the data separates DMs according to the disease type reported in the International Statistical Classification of Diseases and Related Health Problems. **(b)** Same as **a** but for a matrix of pairwise Jaccard distances between DMs. The concordance scores (C-score) over Dim 2 for these projections are 0.764 and 0.752, respectively.

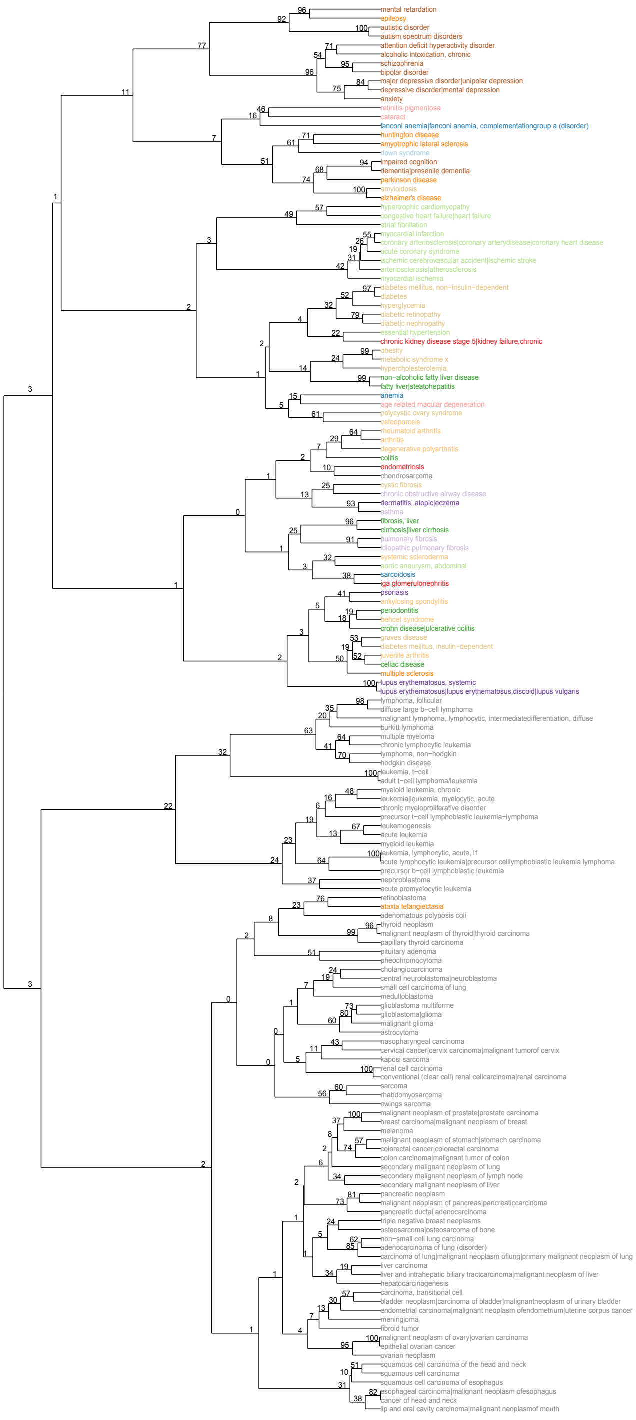

Figure S5: Hierarchical clustering of DMs Unsupervised classification of DMs via hierarchical clustering with Ward's linkage. The numbers next to each node in the dendrogram are percentages indicating the support for the node. These supports were computed by multiscale bootstrap resampling and high values mean that there is strong evidence for the cluster to the of the node, even if any other DM is excluded from the analysis. Colour code as in Fig. S4.

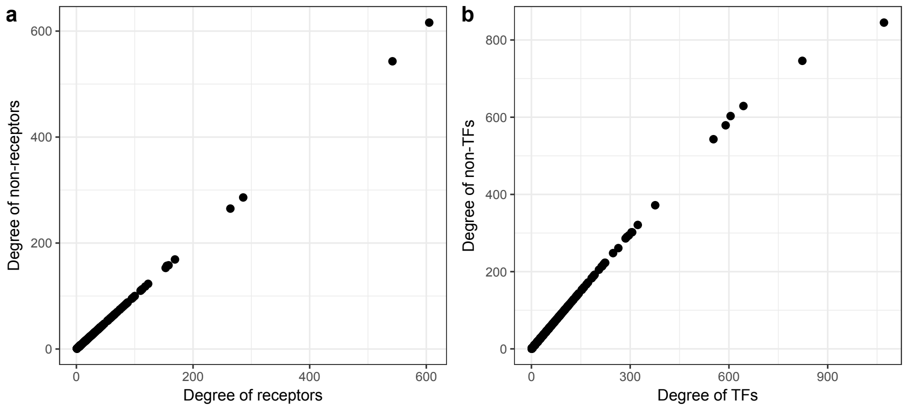

Figure S6: Pools of non-receptor and non-TF proteins A list of **(a)** non-receptors and **(b)** a list of non-TFs were assembled from the hPIN, such that their degree distributions were similar to that of actual receptors and transcription factors, respectively.

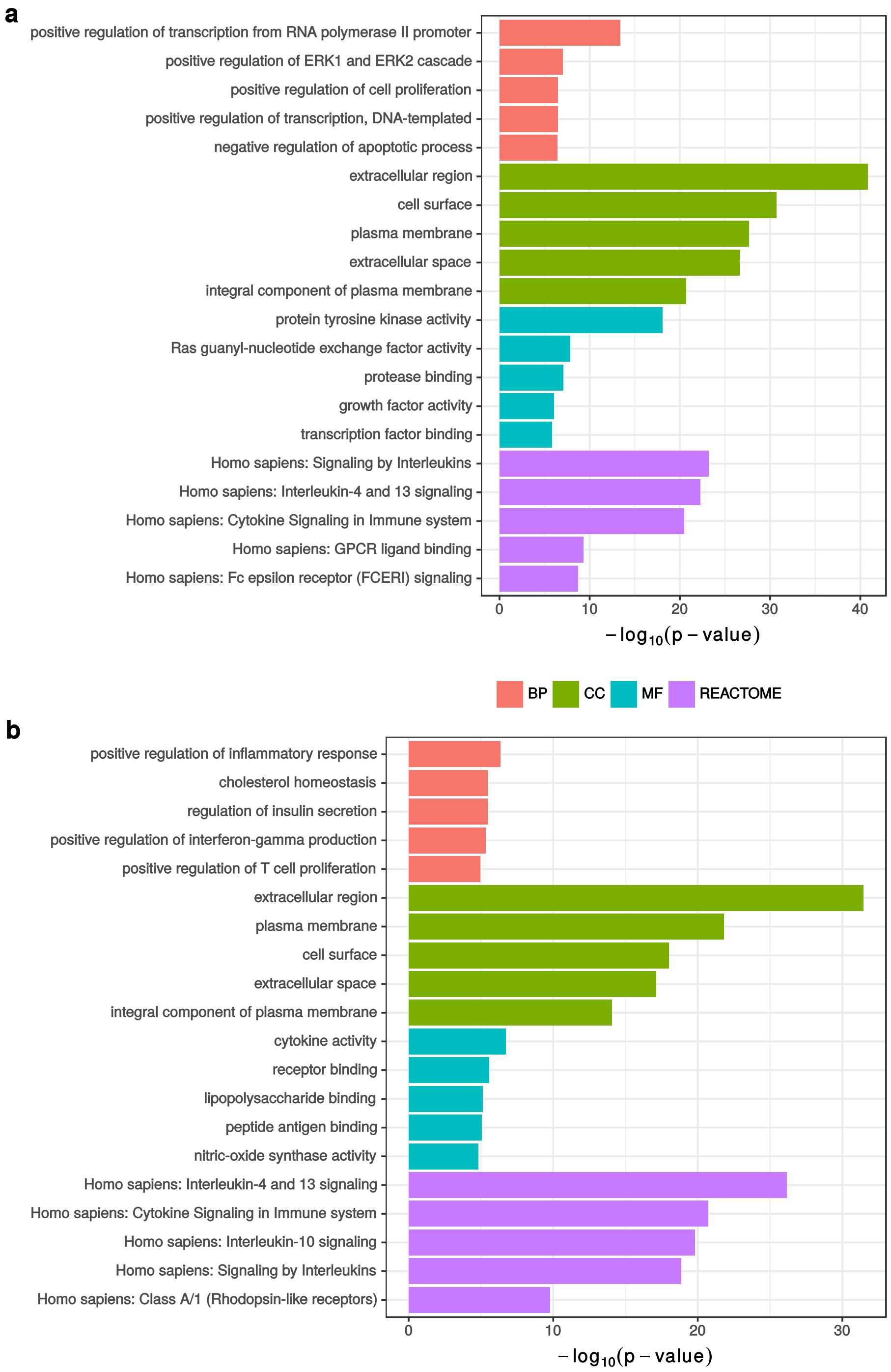

Figure S7: GO and REACTOME functional enrichment analyses of impactful DMs (a) Proteins associated with DMs whose members cause a significant impact on network navigability are receptors or ligands (see CC terms) whose failure affects transcription and apoptosis (see BP terms). **(b)** Proteins associated with DMs whose members don't cause a significant impact on network navigability are receptors or ligands (see CC terms) whose failure affects inflammatory responses and cell homeostasis (see BP terms). BP: Biological Process, CC: Cellular Compartment, MF: Molecular Function.

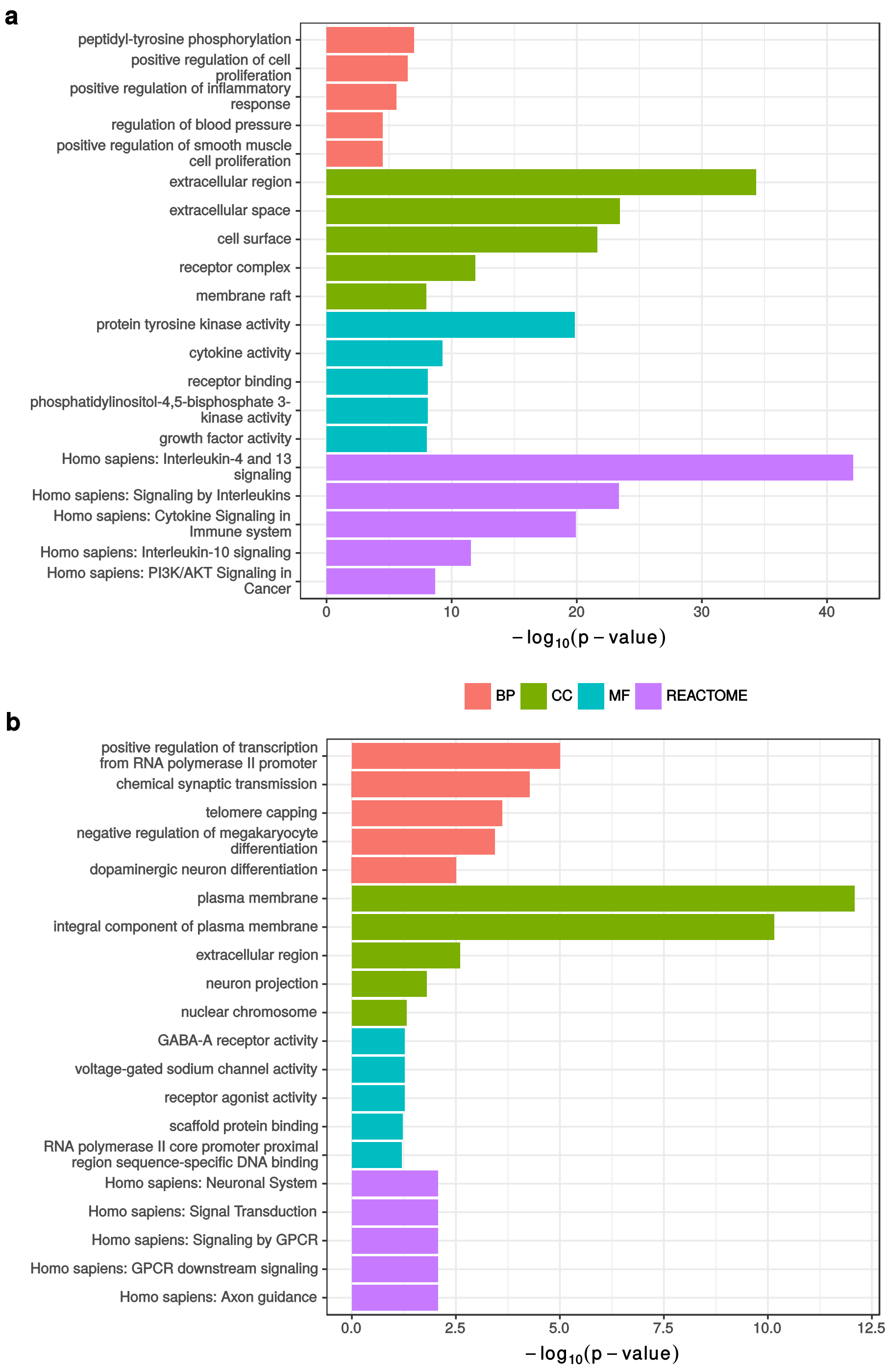

Figure S8: GO and REACTOME functional enrichment analyses of frequent faulty proteins (a) Frequently used faulty proteins are enriched in enzymatic and receptor activities. **(b)** Infrequent faulty proteins have more heterogeneous biological functions. BP: Biological Process, CC: Cellular Compartment, MF: Molecular Function.

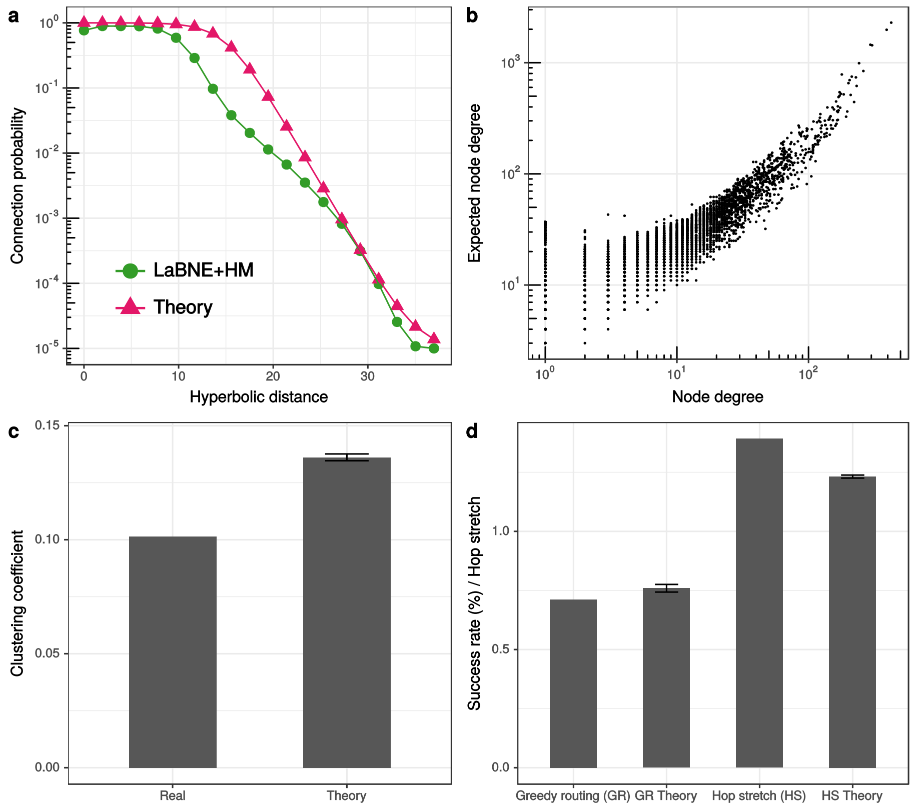

Figure S9: Topological evaluation of the HINT embedding. The quality of the hyperbolic embedding of the HINT protein network was assessed with four different criteria: **(a)** Empirical connection probabilities (LaBNE+HM) agree with those predicted by the Popularity-Similarity model (PSM, Theory). **(b)** Expected node degrees $\langle k_i \rangle = \sum_{j \neq i} p_{ij}$ are similar to actual node degrees. $p_{ij} = 1/[1 + e^{(x_{ij} - R)/2T}]$ is the probability that node i forms a link with node j and depends on the hyperbolic distance x_{ij} between them. R is the radius of the hyperbolic disc containing the network and T is the network temperature. **(c)** The clustering coefficient of HINT is similar to the clustering of artificial networks generated with the PSM, using the same topological properties of HINT (the average across 10 PSMs is reported, errors bars correspond to standard deviations). **(d)** Greedy routing success rates and hop stretches are similar those achieved in artificial networks generated with the PSM, using the same topological properties of HINT (the average across 10 PSMs is reported, errors bars correspond to standard deviations).

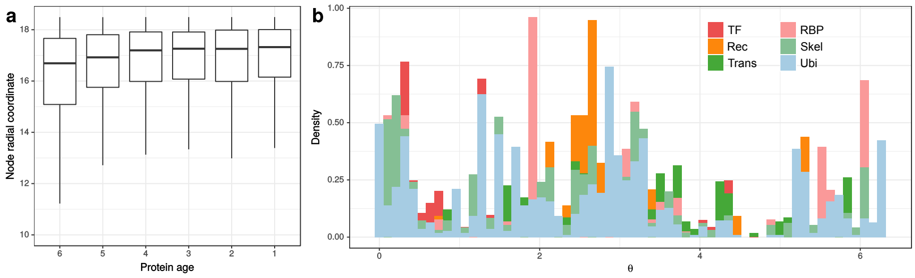

Figure S10: Biological evaluation of the HINT embedding. **(a)** Old proteins tend to be close to the centre of hyperbolic space, while young proteins lie on its periphery. Age groups correspond to human proteins also present in 6: Cellular organisms, 5: Metazoa, 4: Chordata, 3: Mammalia, 2: Euarchontoglires, 1: Primates. **(b)** The inferred angular coordinates of proteins capture the functional organisation of the cell.

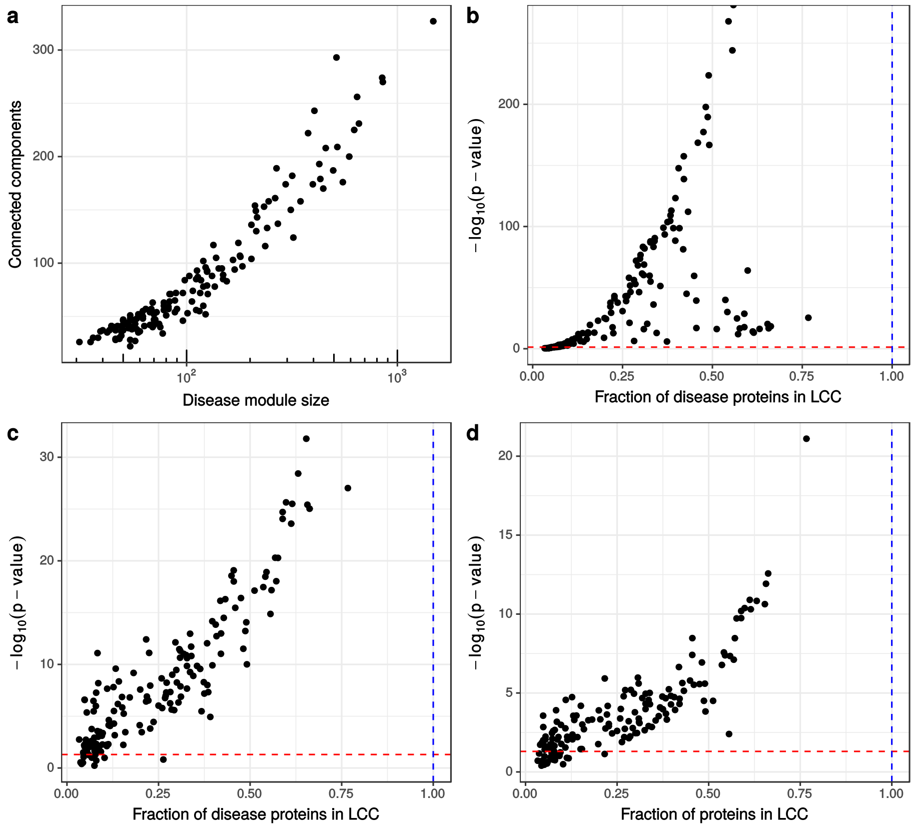

Figure S11: Topology and geometry of DMs in HINT. **(a)** The 157 studied DMs are split into several connected components in HINT. **(b)** The LCC size of each DM was compared with a random distribution of LCC sizes via a z-test. **(c)** The average of the shortest paths from each disease protein to its topologically-closest other DM member ($\langle d_s \rangle$) was compared with random expectation via a z-test. **(d)** The average of the hyperbolic distances from each disease protein to its geometrically-closest other DM member ($\langle d_H \rangle$) was compared with random expectation via a z-test. The red lines correspond to the significance level $\alpha = 0.05$ and the blue ones to the situation in which all proteins associated with a disease form a single connected component.

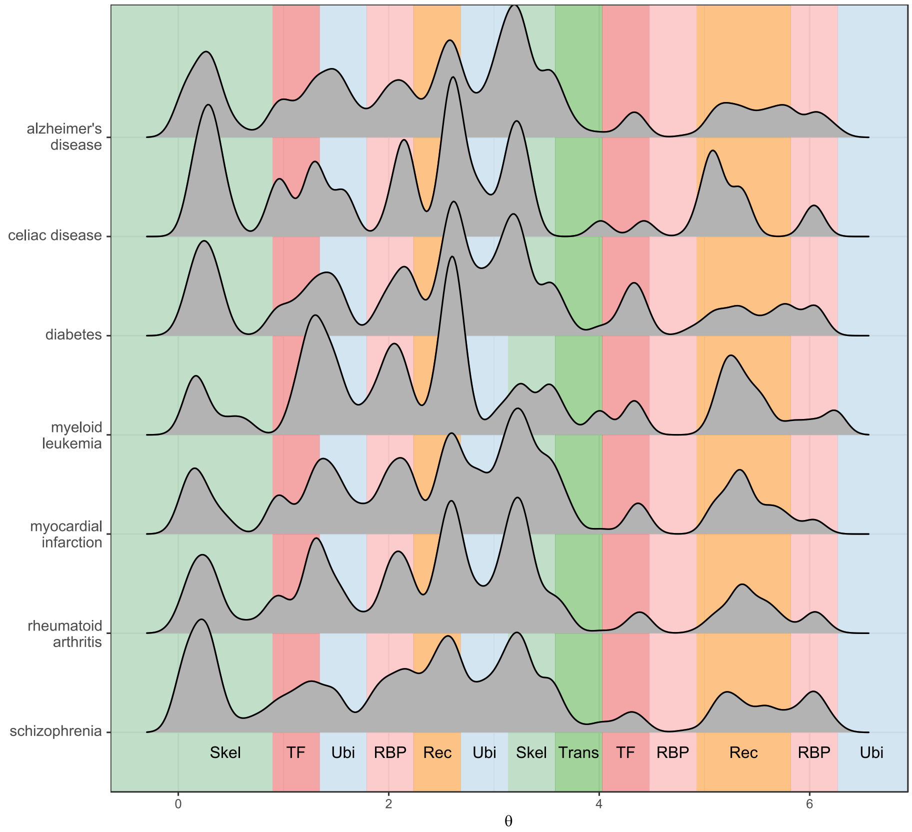

Figure S12: Disease modules in HINT split into functionally distinct submodules. Angular distribution of disease proteins associated with 7 illnesses from our gene-disease association dataset. The coloured backgrounds indicate that one of 6 considered protein classes is over-represented in that angular range (Ubi: proteins involved in ubiquitination/proteolysis, TF: transcription factors, RBP: RNA-binding proteins, Trans: transporters, Skel: constituents of the cytoskeleton, Rec: receptors).

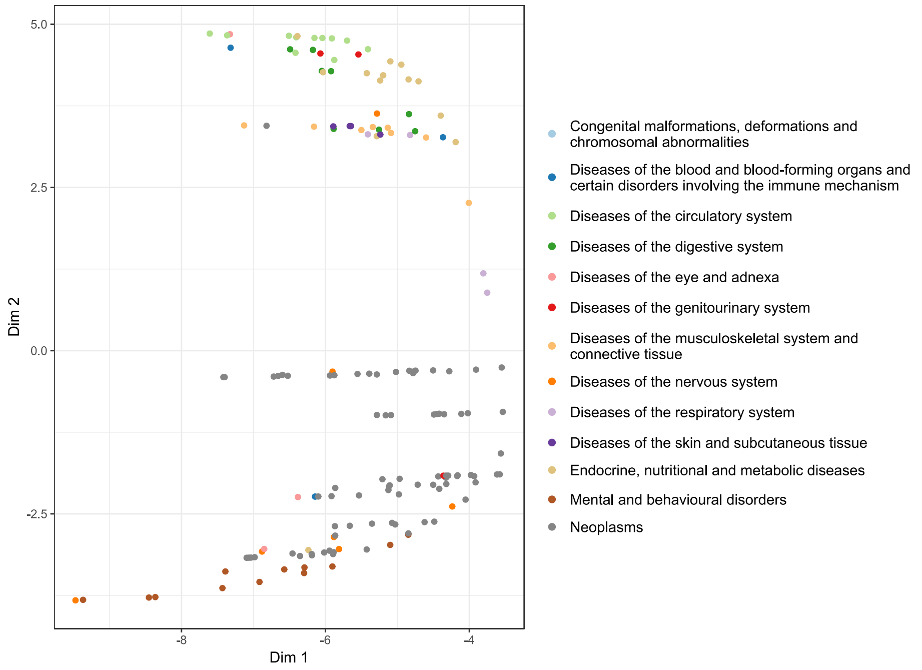

Figure S13: Distance-based clustering of disease modules in HINT. ncMCE, a nonlinear dimensionality reduction algorithm, was applied to the matrix of pairwise hyperbolic distance-based DM separations. The resulting two-dimensional projection of the data separates DMs according to the disease type reported in the ICD-10.

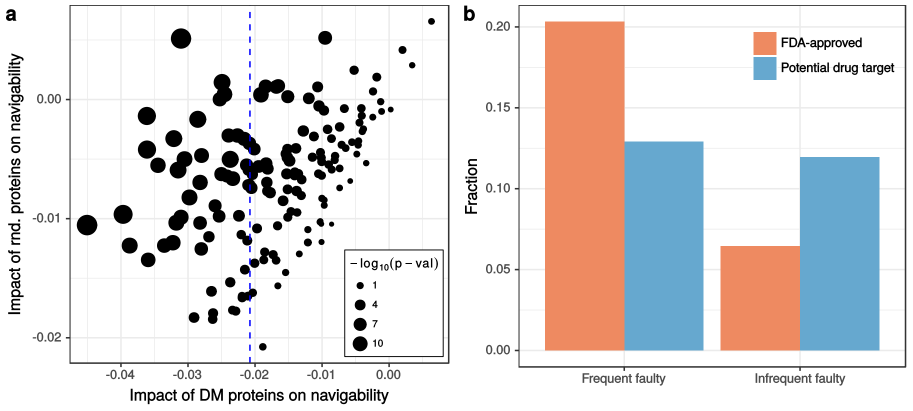

Figure S14: Disease proteins affect the navigability of the HINT network. (a) Impact on GR efficiency (navigability) dealt by faulty proteins sampled from the set of proteins associated with a DM or from a pool of proteins with similar degrees. The blue dashed line indicates the value of the highest impact (i.e. the most negative) from the latter case. **(b)** Frequently used faulty proteins are more likely to be FDA-approved drug targets or potential drug targets than infrequent faulty proteins.
